# Novel Biocomposite Engineering and Bio-Applications

**DOI:** 10.3390/bioengineering5040080

**Published:** 2018-09-28

**Authors:** Gary Chinga-Carrasco

**Affiliations:** RISE PFI, Høgskoleringen 6b, 7491 Trondheim, Norway; gary.chinga.carrasco@rise-pfi.no; Tel.: +47-908-36-045

The engineering and utilization of biocomposites is a research field of major scientific and industrial interest worldwide. Biocomposites include materials composed of at least two components with a distinct morphology and chemistry, where at least one component is bio-based. Furthermore, biocomposites can be classified into different areas depending on their specific application. Hence, in this special issue, various research groups were invited to contribute and cover several aspects and applications of biocomposite materials, spanning from solid biocomposites for structural applications, films such as antimicrobial barriers, to soft biocomposites for specific biomedical purposes, e.g., drug delivery and scaffolds for tissue regeneration.

During recent years, bio-based polymers have attracted major attention due to growing environmental concerns, e.g., ocean littering. There are various types of bio-based polymers or bioplastics, including durable, compostable, and biodegradable materials, suited for specific applications [1,2]. Bioplastics such as polylactic acid (PLA) can be derived from a series of biomass resources, including corn, sugar beet, and sugar cane. PLA has several advantages and can degrade in industrial compostable conditions. The mechanical properties of PLA may be improved by the addition of cellulose nanofibres, provided that the interfacial adhesion between the nanofibers and the PLA matrix is optimized. This has been addressed by Immonen et al. [3], where various types of cellulosic materials were tested for the reinforcement of PLA. Both the type of cellulosic material and the additives used for better dispersion in the PLA matrix were found to affect the mechanical properties of the biocomposites. 

PLA is a versatile polymer, which can be used for encapsulation purposes with a range of applications, e.g., within cosmetics. The study conducted by Kesente et al. [4], demonstrated the potential of PLA nanoparticles for encapsulating olive leaves’ extract. The loaded nanoparticles were incorporated in cosmetic formulations. The encapsulated olive leaves’ extract showed a higher stability compared to pure extract and opens the possibility to better exploit the potential of plant and herb extracts rich in natural antioxidants. This can be used in topical formulations to enhance the skin’s endogenous protection system against oxidative damage [4]. Furthermore, encapsulation is an attractive approach for various products [5]. The potential of cyclic water-soluble oligosaccharides (cyclodextrins) to encapsulate oregano essential oil was demonstrated [5]. The authors suggested a range of applications for the cyclodextrin capsules, such as in the preparation of films for active packaging of food products, in personal care products, and for the improvement of their properties, e.g., antioxidant and antimicrobial [5].

Biopolymers such as PLA and polyhydroxyalkanoates (PHA) are polyesters which have a range of applications within biomedicine, e.g., for the fabrication of artificial tissue or scaffolds for bone regeneration [6]. This is facilitated by three-dimensional (3D) printing technology. 3D printing has gained major attention in recent years due to the capability of technology to create personalized and complex devices. Fused deposition modeling (FDM) is perhaps the most used technology for 3D printing of PLA-based constructs with potential applications within biomedicine, e.g., scaffolds and prosthetics. Additionally, PHA is also printable by FDM technology. According to the authors, PLA and PHA are suitable materials for in vivo applications due to their biocompatibility, biodegradability, good mechanical strength, and processability [6]. 

Natural polymers such as bacterial cellulose have been a focus for years and a range of applications have been proposed, including food packaging, biomedical devices, cosmetics, and as a barrier for degraded paper restoration. Bacterial cellulose consists of pure cellulose, with high crystallinity and a high degree of polymerization. The composition of bacterial cellulose can be modified with additional polysaccharides such as xylans, to form biocomposites with tailored properties [7]. Comprehensive characterization and understanding of the effect of xylan on the properties of bacterial cellulose were performed and shed more light on the potential of the formed biocomposite material [7]. Cotton is another cellulose-rich fiber applied to form textiles. Additionally, cotton fabrics can be functionalized with metal-organic frameworks to form substrates for the filtration of wastewater, allowing photocatalytic activity, decontamination, and micropollutant degradation as demonstrated by Schelling et al. [8]. Surface functionalization of materials to introduce new properties is also promising for biomedical devices. Villegas et al. [9] reported an interesting approach where bioceramics were modified with a lysine amino acid with a zwitterionic function that provides resistance to bacterial biofilm formation. The modified biomaterial was tested against *E. coli* and *S. Aureus*, thus demonstrating the effect of the surface modification on limiting the biofilm formation of the assessed microorganisms. 

Polymers intended for biomedical use (biomaterials) are a timely topic of research. Biomaterials have potential in e.g., regenerative medicine, as drug delivery vehicles, and wound dressings, provided that the biomaterials are biocompatible with the human organism. Cellulose and chitosan are two major natural polymers that have been combined to design biocomposite hydrogel beads proposed as scaffolds for bone tissue engineering [10]. The study demonstrated the potential of natural polymers and a facile chemical approach to design hydrogel beads that were tested for cytocompatibility and cell attachment, important initial aspects to consider if the biomaterial is intended for tissue engineering applications. In addition to natural polymers to fabricate scaffolds for bone tissue engineering [10], repair of bone tissue using hypertrophic cartilage grafts has been demonstrated in this issue [11]. The authors explored the development of a devitalized hypertronic cartilage matrix in amounts that were clinically relevant and assessed its effect on chondrogenic differentiation in vivo [11]. 

Tissue engineering includes a series of biomaterials and applications with the potential to improve people’s quality of life, of which the regeneration of neural tissue is a specific example. Hence, biomaterials that counteract neurodegenerative disorders by repairing damaged tissue and promoting the growth of healthy tissue pose great societal benefits. Biocomposite scaffolds are necessary to mimic the properties of healthy tissue. In this issue, self-assembled nanoribbon combined with conductive polymers were the basis to form biocomposite scaffolds which promoted growth and proliferation of cortical cells and axonal outgrowth [12]. Provided that the scaffolds are biodegradable to promote cell proliferation and that biocompatibility is secured, the approach seems promising for neural tissue engineering. Additionally, biomaterials based on proteins are interesting for biomedical devices due to their biodegradability and biocompatibility [13]. This paper provides a clear example of advanced bioengineering processes for the biosynthesis of proteins containing non-canonical amino acids. The chemical functionalities of proteins can thus be tailored, expanding their characteristics and applications within biomaterial science [13]. Importantly, the use of biomaterials as scaffolds for tissue engineering requires a thorough understanding of the mechanical properties of the biomaterials and how these properties affect cellular behavior such as proliferation and differentiation. It is thus of great importantance to have adequate methods to measure and understand the cell-cell interactions and the mechanotransduction between cells and the surrounding matrix. These physiological processes were extensively reviewed by Zhang et al. [14], focusing on the detailed understanding of the mechanosensory responses of cells by using e.g., cell traction force microscopy techniques. Assessment of the cascade of signals involved in mechanotransduction in 3D microenvironments is expected to facilitate the design of tailored scaffolds for tissue engineering [14].

Finally, during recent years there has been an extensive development of bio-based materials intended for biomedical applications (e.g., scaffolds for tissue engineering or wound dressings). Although these bio-based materials are derived from natural biomass resources and are generally considered to be non-toxic, some of the materials have nano-dimensions and surface chemistries that differ from the original and natural state. It is thus of utmost importance to ensure that such nano-dimensions and surface modifications do not compromise the biocompatibility. These aspects, in addition to relevant endpoints, should be evaluated in every case to secure safety and human health [15]. 
